# Flexible Piezoelectric Sensor for Real-Time Comprehensive Cardiovascular Monitoring

**DOI:** 10.1021/acsaelm.5c02070

**Published:** 2026-01-02

**Authors:** Nathan Zhang, Sun Hwa Kwon, Lin Dong

**Affiliations:** † Department of Electrical and Systems Engineering, 6572 University of Pennsylvania, Philadelphia, Pennsylvania 19104, United States; ‡ Department of Mechanical and Industrial Engineering, 5965 New Jersey Institute of Technology, Newark, New Jersey 07102, United States

**Keywords:** Piezoelectric, biosensors, flexible electronics, signal processing, cardiovascular monitoring

## Abstract

Wearable cardiovascular monitoring requires sensitive sensors and body-conforming electronics for reliable and automatic signal processing. Here, we present a fully self-contained platform that integrates a piezoelectric nanofibrous sensor with a custom flexible printed circuit board (PCB) for on-body charge amplification and filtering. Fabricated on a polyimide substrate with an ultralow bias current amplifier, the PCB preserves high-impedance piezoelectric signals for on-body signal processing. Electromechanical tests verified stable, linear performance improved by thermal annealing. System-level evaluation showed robust operation during cardiovascular monitoring, capturing radial, carotid, and seismocardiogram signals and extracting key cardiac parameters, demonstrating its potential as a practical, comprehensive, and wearable biosensing solution.

## Graphical abstract

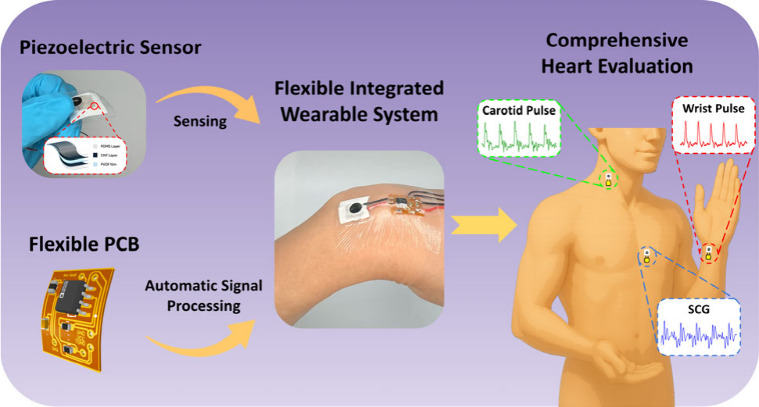

Flexible and wearable electronics can transform personal healthcare by shifting monitoring from brief clinic visits to continuous, real-time tracking of physiological signals.^1,2^ Recent work has advanced flexible sensors and compliant substrates that allow devices to better conform to the human body. However, many research prototypes still contain rigid components such as inflexible housing, interconnects, or batteries, which reduce flexibility, decrease comfort, and restrict use to laboratory settings.^1^ As a result, there remains a substantial gap between academic prototypes and fully self-contained, wearable tools that are suitable for everyday life. Bridging this gap requires two key advances: (1) a physically compliant and self-powered sensor for long-term use and (2) integrated, lightweight, and flexible electronics for on-body signal processing to eliminate rigid components.

Among the available transduction mechanisms, piezoelectric materials are ideal for flexible wearable electronics in the medical landscape as they convert subtle mechanical deformations from biophysiological signals such as arterial pulse waves and chest wall vibrations into measurable electrical outputs. While piezoelectric ceramics (such as lead zirconate titanate (PZT)) and single crystals (such as Lead Magnesium Niobate-Lead Titanate (PMN-PT)) offer high sensitivity, their rigidity and brittleness make them poorly suited for skin-contact applications.^2^ In contrast, piezoelectric polymers offer exceptional mechanical compliance, and their piezoelectric properties can be significantly enhanced through fabrication techniques or composite material formulations, such as the inclusion of nanoparticle composites or the design of core–shell nanofibers, all while bending and stretching over soft tissues without compromising structural integrity.^3–6^ Among piezoelectric polymers, poly­(vinylidene fluoride) (PVDF) is commonly utilized due to its favorable electromechanical properties and processability. Its copolymer poly­(vinylidene fluoride-trifluoroethylene) (P­(VDF-TrFE)) exhibits a higher fraction of the piezoelectrically active β-phase, which enhances polarization stability and results in improved charge generation efficiency when compared to PVDF.^3^ It can also crystallize directly into the piezoelectrically active beta phase without requiring mechanical stretching, leading to a higher and more stable remanent polarization and enhanced piezoelectric coefficients.^7–9^ Hence, the inherent lightweight, fatigue-resistant, and chemically inert nature of P­(VDF-TrFE) ensures safe and reliable skin contact for wearable applications.^10^ Various fabrication methods are compatible with piezoelectric polymers, including solution casting, spin coating, and electrospinning.^2,11,12^ Among these, electrospinning is particularly advantageous for wearable applications because it produces nanofibrous membranes with high surface area, intrinsic porosity, and exceptional mechanical compliance.^13^ This process in situ induces molecular chain alignment and promotes β-phase crystallinity, enhancing piezoelectric performance and yielding durable, sensitive, and conformable membranes well suited for skin-worn sensors.

However, when deformed, piezoelectric polymers produce small, high-output impedance signals that require specialized electronics.^14^ Miniaturized systems often reply on rigid printed circuit boards (PCBs), whose mechanical inflexibility introduces motion artifacts and degrades signal quality.^15^ Flexible PCBs overcome these limitations by providing reliable electrical interconnections in thin mechanically compliant formats; they can bend and twist while maintaining stable electrical performance and are therefore widely used in space- and weight-constrained applications across industries ranging from consumer electronics to aerospace. In wearable systems, flexible PCBs enable thin, lightweight, and body-conforming signal-processing modules.^2^ Yet, few studies have explored their integration with high-impedance piezoelectric sensors, despite the potential of this pairing to minimize mechanical stress, enhance comfort, and enable fully integrated systems optimized for long-term wear.^16,17^ In particular, integration with custom flexible PCBs offers a key advantage over rigid electronics by reducing motion artifacts caused by mechanical mismatch with the skin and improving user comfort during continuous wear.^15^

In biophysiological monitoring, cardiovascular assessment is particularly crucial as it is a significant indicator of cardiovascular diseases (CVDs). Subtle changes in cardiac function and vascular dynamics can serve as early indicators of disease progression, enabling timely diagnosis and intervention.^17^ To address the challenges outlined above, this work presents a fully integrated, flexible, and wearable system for cardiovascular assessment, closing the gap between laboratory demonstrations and practical on-body applications. The platform integrates a self-powered, flexible piezoelectric sensor fabricated from electrospun P­(VDF-TrFE) nanofibers with a custom flexible PCB for on-body signal processing. As shown in Figure 1, the flexible system acquires radial, carotid, and seismocardiogram (SCG) signals from the wrist, neck, and chest, respectively. The primary innovation of this work is not the material itself but the seamless integration of a high-performance, nanofibrous piezoelectric sensor with a custom-designed, body-conformable signal conditioning circuit into a single, untethered platform. This integration enables, for the first time, multisite, high-fidelity cardiovascular monitoring (wrist, neck, and chest) using a self-contained wearable device, bridging the gap between isolated sensor development and practical holistic physiological assessment.

**Figure 1:**
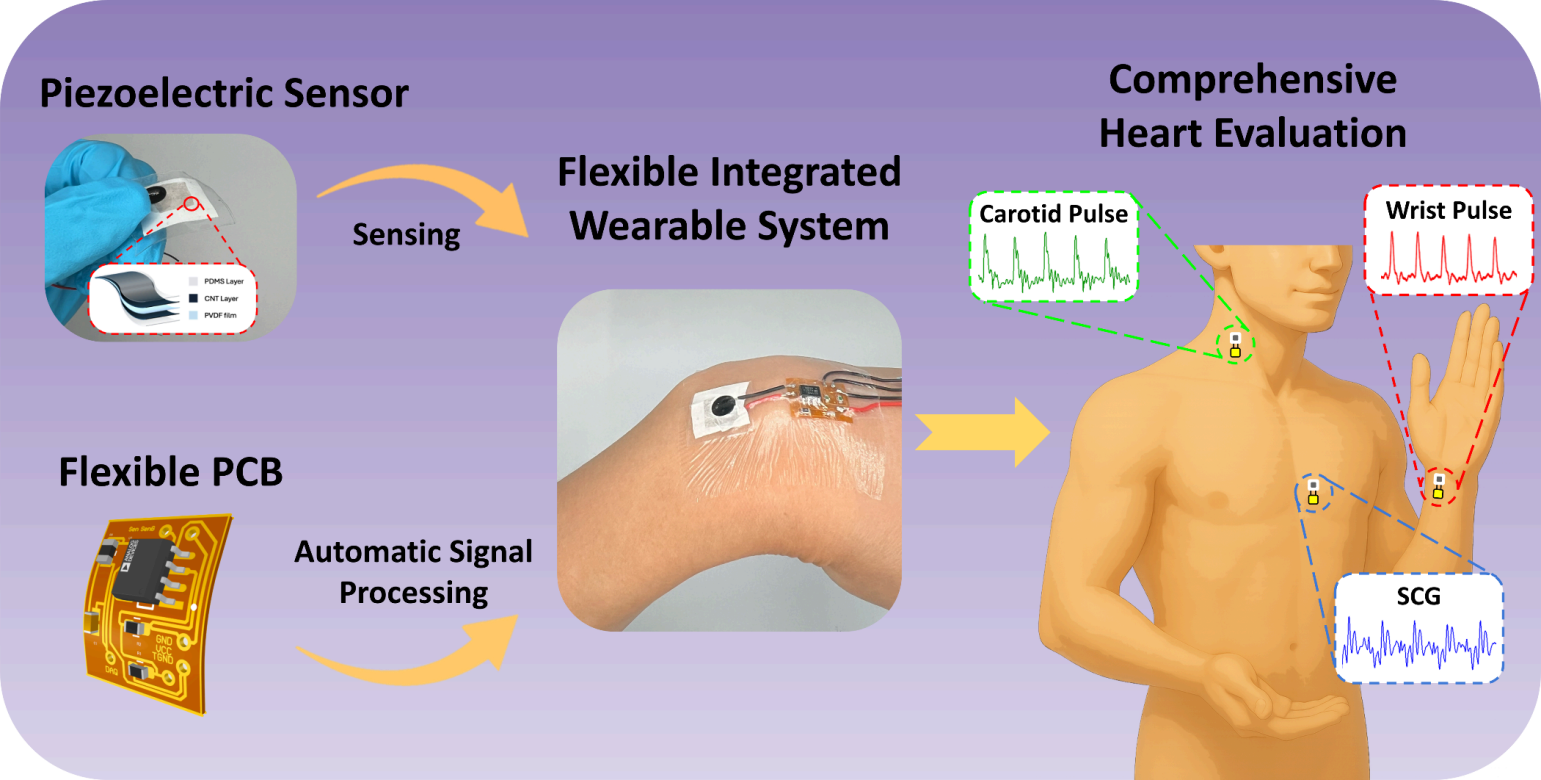
An overview of the integrated, flexible, wearable piezoelectric system for cardiovascular monitoring.

The development of this integrated system begins with the fabrication of the high-performance piezoelectric sensor, which is the core component of the platform. As illustrated in Figure 2, P­(VDF-TrFE) nanofibers were fabricated via the electrospinning (Figure 2A). In this process, a high-voltage electric field was applied to an 18 wt % P­(VDF-TrFE) solution, initiating the formation of a polymer jet that was mechanically stretched as it traveled toward the rotating mandrel. The combined influence of the electric field induced elongation and mechanical stretching to generate tensile stress that aligned the molecular chains.^18^ This in situ alignment, together with intrinsic poling during electrospinning, facilitated the transformation of the polymer into the piezoelectrically active β-phase, the dominant contributor to the material’s electromechanical response.^19^

**Figure 2:**
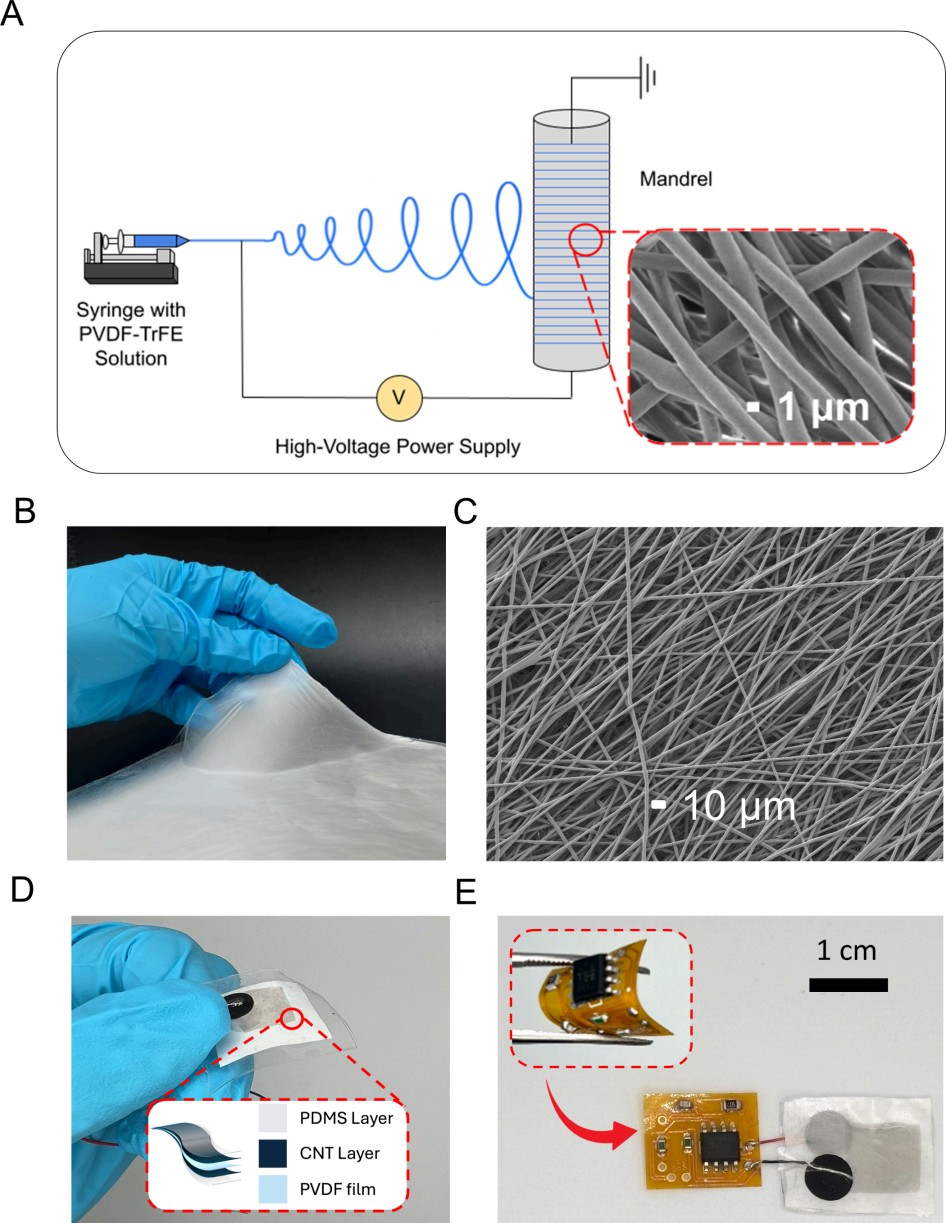
(A) Schematic of the electrospinning technique used to fabricate aligned P­(VDF-TrFE) nanofibers. (B) A digital image of the resultant flexible electrospun nanofiber membrane. (C) SEM image showing the uniform, nonwoven morphology of the aligned nanofibers. (D) A digital image of the assembled multilayered piezoelectric sensor. (E) A digital image of the fully integrated wearable device.

The electrospinning process produces a flexible membrane of aligned P­(VDF-TrFE) nanofibers (Figure 2B), which then undergoes thermal annealing to optimize piezoelectric performance. Annealing enhanced the β-phase crystallinity, improving both sensor sensitivity and electromechanical stability.^19^ Fourier-transform infrared spectroscopy (FTIR) confirmed an increase in β-phase fraction from 73% to 81% after annealing (Supporting Information, Figure S1),^20^ while X-ray diffraction (XRD) analysis showed an increase in overall crystallinity from 74.1% to 84.7% (Supporting Information, Figure S2). Morphological characterization using Scanning Electron Microscopy (SEM) further validated the fabrication process. As shown in Figure 2C, the electrospinning process produced a well-aligned nanofiber network with an average diameter of 1.56 μm, confirming a uniform fiber formation suitable for high-performance piezoelectric sensing.

The flexible piezoelectric sensor features a multilayered architecture engineered for high sensitivity and wearer comfort (Figure 2D). A functional P­(VDF-TrFE) nanofibrous layer is encapsulated between two flexible conductive electrodes and sealed with biocompatible outer layers. Single-walled carbon nanotubes (CNTs) were selected as the electrode material because unlike conventional metal films (e.g., gold or silver) that tend to crack under strain, CNT networks maintain electrical integrity during deformation. Their interconnected, mesh-like morphology provides excellent electrical conductivity while accommodating bending and stretching inherent to wearable use, ensuring stable signal acquisition during movement.^21,22^ For the encapsulation material, biocompatible and flexible polydimethylsiloxane (PDMS) was used. The CNT electrodes were fabricated by transferring CNT mats onto semicured PDMS films via a solvent-free contact pressing method, which preserves the dense CNT network and yields uniform, highly conductive films, Finally the full sensor was assembled through a layer-by layer process that integrates the CNT electrodes, the P­(VDF-TrFE) nanofiber layer, and the PDMS encapsulation into a single flexible device.

Next, to enable fully untethered operation, we developed a lightweight, skin-conformable electronic module that integrates directly with the sensor, eliminating the reliance on external instrumentation (Figure 2E). The on-body signal conditioning circuit functions as a charge amplifier, converting the small charge generated by the high-impedance piezoelectric sensor into a measurable voltage. An analog device AD548 operational amplifier was selected for its ultralow input bias current, which is essential for preserving weak charge signals from the sensor without introducing additional loading (Supporting Information, Figure S3). The entire circuit is implemented on a 25 μm-thick polyimide flexible PCB, allowing self-contained, body-conforming acquisition of biomechanical signals by converting sensor charge to a stable filtered voltage.

After the multilayer sensor is constructed, it is important to distinguish the primary sensing mechanism from potential signal artifacts. The symmetric, fully encapsulated architecture (PDMS/CNT/P­(VDF-TrFE)/CNT/PDMS) is intentionally designed to suppress triboelectric contributions; any charge generated at one interface would be largely canceled by an equivalent but opposite interface. Furthermore, the active layer, P­(VDF-TrFE), is a well-established ferroelectric polymer whose strong piezoelectric response emerges only after electrical poling aligns its molecular dipoles. Thus, the consistent and repeatable signals produced during deformation can be attributed to strain-induced dipole moment changes within the poled piezoelectric core. Further experimental electromechanical testing will validate this interpretation.

To validate its sensing performance, Figure 3 demonstrates a series of electromechanical characterization tests that were conducted using a shaker-based platform capable of applying tunable forces and excitation frequencies. A key objective of this analysis was to evaluate the effect of thermal annealing on sensor performance. Compared with the nonannealed sensor, the annealed sensor produced ∼40% higher peak-to-peak voltage (Figure 3A) and demonstrated substantially improved sensitivity (101.2 mV/N vs 47.6 mV/N) with higher linearity (Figure 3B). These results confirm that thermal annealing significantly enhances the piezoelectric performance of the P­(VDF-TrFE) sensor.

**Figure 3:**
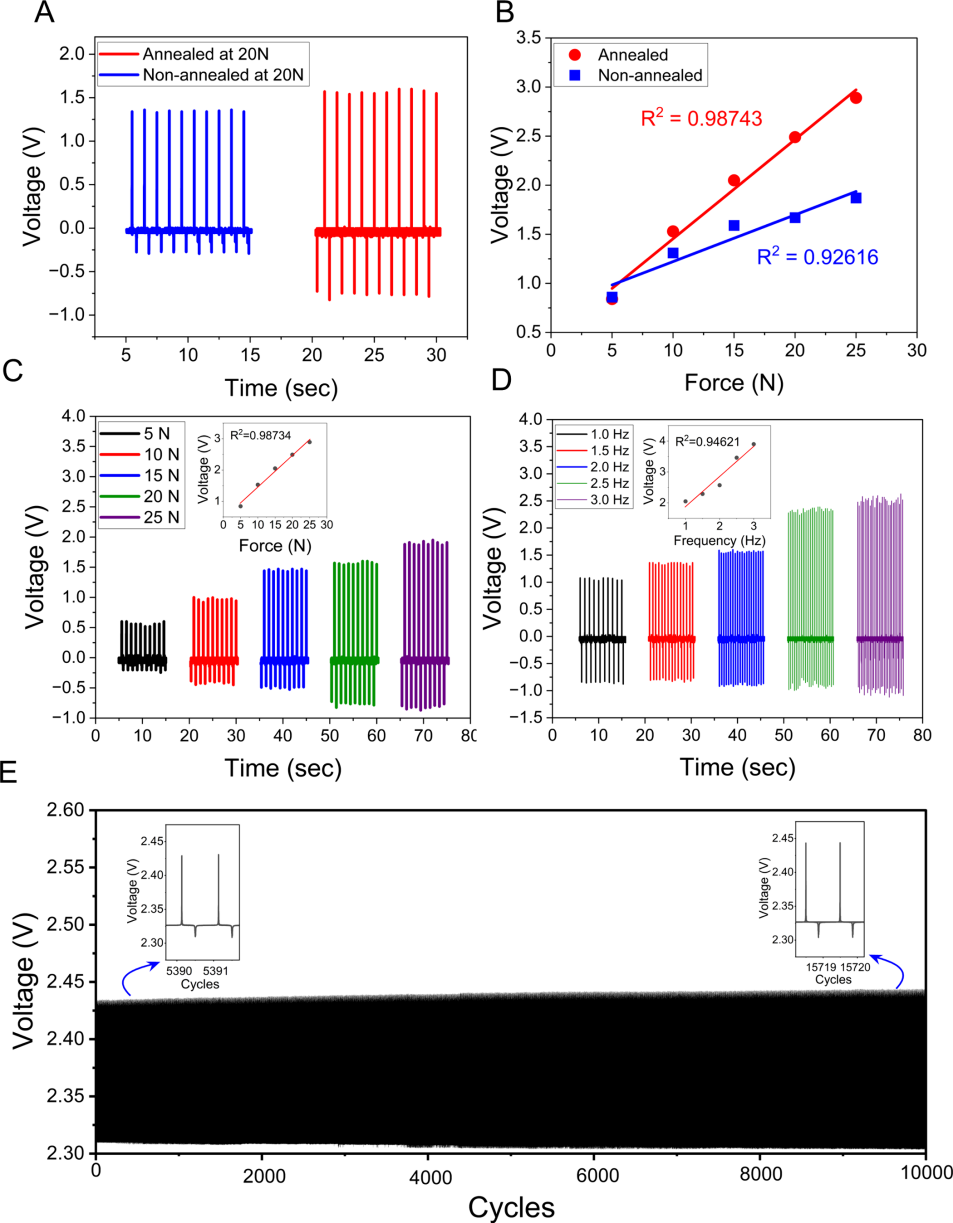
(A) Real-time voltage output comparing the annealed versus nonannealed sensor. (B) A plot of the force-voltage relationship of the annealed versus nonannealed sensor. (C) The linear voltage response of the annealed sensor to increasing applied forces. (D) The linear voltage response of the annealed sensor to increasing excitation frequencies. (E) Antifatigue testing of the annealed sensor at 5 N and 1 Hz at 10,000 cycles.

With the efficacy of the annealing process established, the annealed sensor’s response was further characterized under varying mechanical conditions. Under cyclic forces of 5, 10, 15, 20, and 25 N at a constant frequency of 1 Hz, the peak-to-peak voltage increased proportionally with the applied load (Figure 3C), with the inset graph confirming a strong linear relationship (R^2^ = 0.98734). This behavior follows the piezoelectric relationship V = g_33_ F t/A, where g_33_ is the piezoelectric voltage constant.^23^ The sensor’s frequency response was then evaluated by applying excitation frequencies of 1, 1.5, 2, 2.5, and 3 Hz at a constant input force of 20 N (Figure 3D). The voltage output increased with frequency, consistent with the direct piezoelectric effect described by I = d_33_ A dσ/dt, where the rate of stress change (dσ/dt) increases with excitation frequency.^23^ The inset linear fit (R^2^ = 0.94621) demonstrates the sensor’s high fidelity in capturing dynamic signals. This experimental characterization supports the interpretation of a strong piezoelectric response with negligible triboelectric contributions. The sensor’s output voltage increases linearly with excitation frequency (Figure 3D), reflecting a higher rate of stress change (dσ/dt). Such frequency-dependent behavior is a hallmark of the direct piezoelectric effect, where charge generation is proportional to dσ/dt, and is fundamentally distinct from triboelectric signals, which depend on intermittent contact-separation rather than the rate of continuous strain.^24,25^

Finally, the long-term durability and stability of the sensor, both essential for wearable use, were evaluated through repeated mechanical loading. The device withstood 10,000 continuous loading cycles at 5 N and 1 Hz (Figure 3E) without noticeable signal degradation, confirming its robustness for extended physiological monitoring. Furthermore, the consistent signal output across all cycles shows strong material integrity under repeated mechanical stress and indicates that the sensor can reliably function in long-term wear scenarios. To further assess its mechanical resilience under conditions relevant to daily wear, the system also underwent 5,000 bending cycles, during which it exhibited negligible loss in piezoelectric response (Supporting Information, Figure S4). These results together demonstrate that both the P­(VDF-TrFE) sensing layer and the CNT-based flexible electrodes maintain a stable performance under repeated mechanical deformation.

Following electromechanical validation, the integrated system was used to capture cardiovascular signals from volunteers at multiple anatomical sites. The device was adhered to the wrist, neck, and chest to perform a comprehensive, multisite cardiovascular assessment (Figure 4). To evaluate peripheral arterial dynamics, the sensor was first positioned over the radial artery at the wrist (Figure 4A). The real-time piezoelectric response (left graph of Figure 4B, and Supporting Information, Video S1) and the corresponding integrated waveform (right graph of Figure 4B) both clearly capture the radial pulse. The integrated waveform reveals two distinct peaks: P_1_, corresponding to the initial forward systolic pressure wave generated by ventricular ejection, and P_2_, corresponding to the late systolic reflected wave produced by peripheral arterial reflection.^26^ The timing and amplitude of those peaks allow computation of the augmentation index (AIx = P_2_/P_1_), a well-established measure of systemic arterial stiffness typically ranging from 0.4 – 0.6 in healthy young adults.^27^ From the recorded signals, the volunteer’s AIx value was approximately 0.55, indicating a healthy level of arterial flexibility (Supporting Information, Table S1).

**Figure 4:**
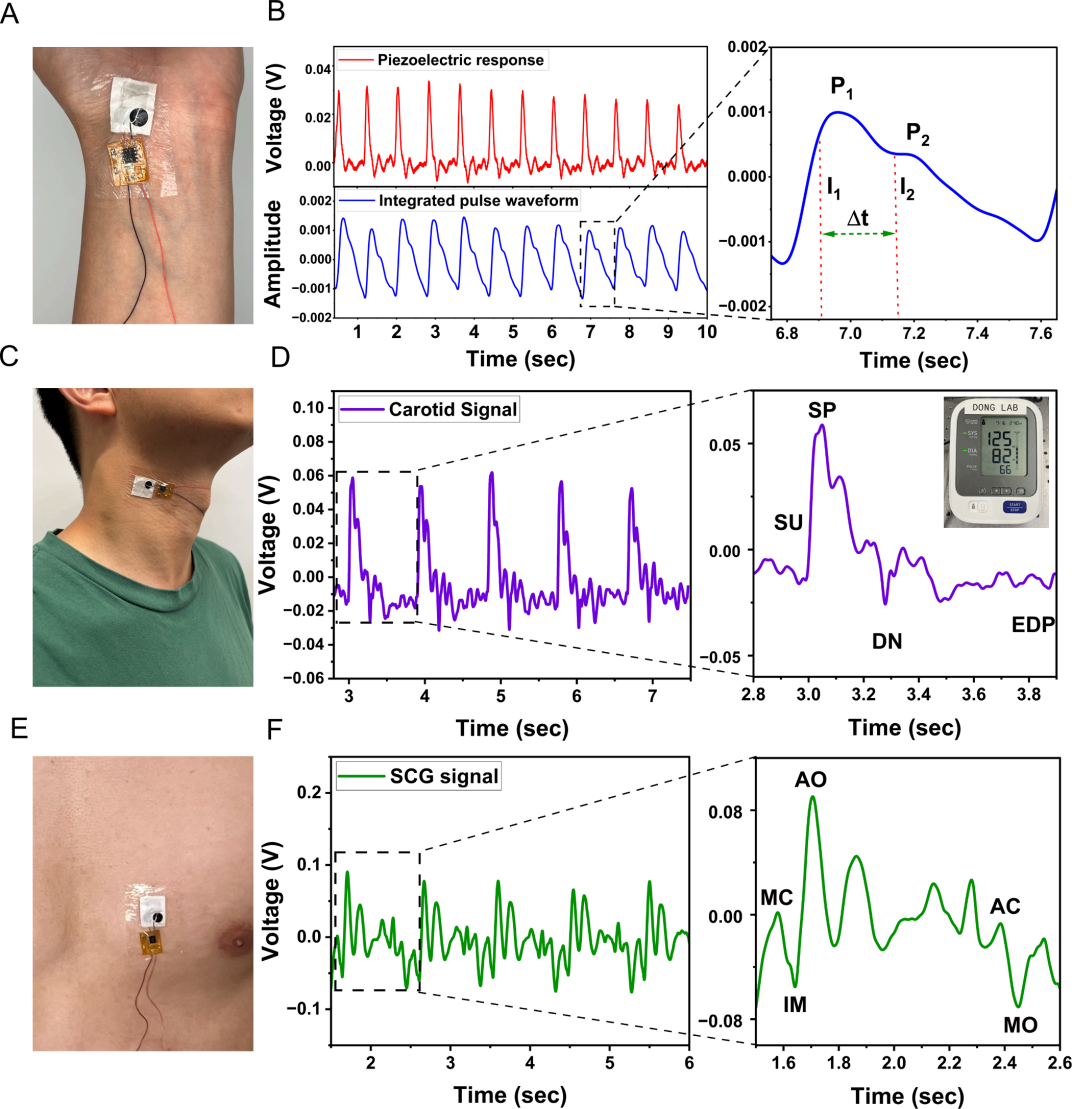
(A, B) The wrist sensor captures the radial pulse wave, with the zoomed-in view detailing the P_1_ and P_2_ peaks. (C, D) The neck sensor records the carotid pulse, showing key morphological features, such as SP and DN. (E, F) The chest sensor provides SCG signals, with the detailed view showing peaks that correspond to mechanical events of the cardiac cycle.

For the assessment of central hemodynamics, the sensor was then placed over the carotid artery on the neck (Figure 4C). The recorded carotid waveform (Figure 4D, and Supporting Information, Video S2) provides a high-fidelity proxy for central aortic pressure, which is a more clinically relevant indicator of cardiovascular risk than peripheral measurements.^28^ The waveform exhibits key diagnostic features, including the initial systolic peak (SP), the preceding systolic upstroke (SU), the dicrotic notch (DN) that marks the closure of the aortic valve, and the end-diastolic pressure (EDP) marking the minimum pressure at the end of diastole just before the next cardiac cycle. Because the morphology of the carotid wave is directly related to blood pressure and arterial stiffness, these features can be used to estimate central systolic and diastolic values. Continuous tracking of SP, SU, DN, and timing intervals can reveal early changes in the cardiac load or progressive vascular stiffening.

Finally, to demonstrate the system’s versatility in capturing subtle cardiac mechanical activity, the sensor was placed on the volunteer’s chest directly over the heart to record seismocardiogram (SCG) signals (Figure 4E). SCG is a noninvasive method of measuring the heart’s mechanical vibration of the thorax wall during the cardiac cycle. As shown in Figure 4F (Supporting Information, Video S3), the recorded SCG signals reveal a complex waveform with clear annotated peaks corresponding to key cardiac events. The systolic phase begins with the mitral valve closure (MC) peak, marking the onset of ventricular contraction and contributing to the first heart sound (S1).^29^ This is followed by the isovolumic moment (IM), during which the ventricular pressure rises rapidly. The subsequent, high-amplitude aortic valve opening (AO) peak reflects the ejection of blood into the aorta, whereas the diastolic phase begins with aortic valve closure (AC) corresponding to the second heart sound (S2), followed by mitral valve opening (MO) that initiates ventricular filling. Separately, SCG signals obtained from the sensor were compared to those obtained from a commercial accelerometer (Supporting Information, Figure S5). The piezoelectric sensor produces cardiac signals on par with those of commercial systems typically used to obtain SCG signals, confirming its high accuracy and reliability as a flexible piezoelectric platform for cardiovascular monitoring.

These annotated peaks enable the extraction of diagnostically meaningful systolic time intervals. The duration between the MC and AO corresponds to the isovolumic contraction time (IVCT), while the interval between the AO and AC indicates the left ventricular ejection time (LVET). Analysis of the recorded SCG waveform yielded an average IVCT of 64 ± 3 ms and an average LVET of 335 ± 16 ms in a female volunteer with self-reported elevated blood pressure (Supporting Information, Table S2). These values are comparable in magnitude to IVCT and LVET intervals reported for healthy adults in large cohort studies, with IVCT lying toward the upper end published ranges.^30^ These timing intervals are direct and highly sensitive mechanical indicators of myocardial contractility; for example, impaired ventricular function prolongs pressure buildup and results in a measurable increase in IVCT.^30^ Collectively, these cardiac-rich signals obtained from the wrist, neck, and chest confirm that the integrated system can reliably capture distinct biomechanical signatures from multiple anatomical sites, demonstrating the feasibility of the proposed multisite wearable platform for comprehensive cardiovascular assessment.

Ultimately, this work presents a flexible, sensitive, and self-powered piezoelectric sensor platform capable of continuous, real-time biomechanical monitoring. The central innovation lies in the seamless integration of a high-performance P­(VDF-TrFE) nanofiber sensor with a custom flexible PCB for on-body signal conditioning, enabling a fully self-contained wearable device that eliminates the need for bulky external equipment. Comprehensive characterization demonstrated linear, reliable, and durable electromechanical performance, allowing high-fidelity signal acquisition from three distinct anatomical sites: the wrist (radial pulse), neck (carotid pulse), and chest (seismocardiogram). This multisite capability provides a holistic view of cardiovascular dynamics and supports timely physiological assessment, highlighting the platform’s potential to advance clinical monitoring and improve outcome for patients with cardiovascular disease.

## Supplementary Material









## Data Availability

Data supporting the findings of this study are available from the corresponding author upon reasonable request.

## Notes

FTIR spectra comparison between annealed and nonannealed P­(VDF-TrFE) nanofibers (Figure S1); XRD comparison between annealed and nonannealed P­(VDF-TrFE) nanofibers (Figure S2); diagrams of the charge amplifier circuit and PCB schematic (Figure S3); antifatigue testing at cyclic bending for 5,000 cycles (Figure S4); tabulated values for P1, P2, and augmentation index (Table S1); tabulated values for IVCT and LVET (Table S2); comparison graph of SCG signals between commercial accelerometer and P­(VDF-TrFE) sensor (Figure S5) (PDF)
Video S1: Real-time wrist pulse signal acquisition (MP4)
Video S2: Real-time carotid pulse signal acquisition (MP4)
Video S3: Real-time SCG signal acquisition (MP4)
