# Supplementary material for: Flexible Piezoelectric Sensor for Real-Time Comprehensive Cardiovascular Monitoring
Source: ACS Appl Electron Mater. 2026 Jan 2;8(1):48–55. doi: 10.1021/acsaelm.5c02070 (PMC12805564; doi:10.1021/acsaelm.5c02070)
Supplement: Supplementary file 1 [file el5c02070_si_001.pdf]

Supporting Information

Flexible Piezoelectric Sensor for Real-Time Comprehensive Cardiovascular Monitoring

Nathan Zhang[1], Sun Hwa Kwon[2], and Lin Dong[2]*

Department of Electrical and Systems Engineering, University of Pennsylvania, Philadelphia, PA, 19104, USA[1]

Department of Mechanical and Industrial Engineering, New Jersey Institute of Technology, Newark, NJ, 07102, USA[2]

***Corresponding author:** Dr. Lin Dong (lin.dong@njit.edu)

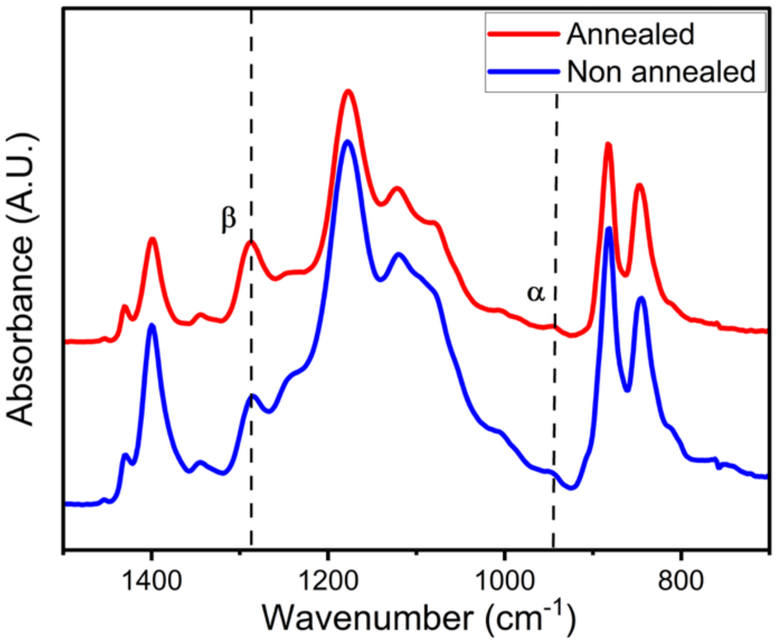

Fig. S1 FTIR spectra of the annealed and the non-annealed P(VDF-TrFE) nanofibers to confirm the crystalline phase transformation of the piezoelectric nanomaterial resulting from the thermal annealing process. The graph compares an annealed sample (red line) with a non-annealed sample (blue line). The prominent absorption band at approximately 1280 cm^{-1} is characteristic of the piezoelectric β-phase, while the peak at approximately 975 cm^{-1} corresponds to the non-polar α-phase [1]. The annealing process increased the β-phase fraction from 73% to 81%, confirming a successful conversion from the non-polar α-phase to the highly polar piezoelectric β-phase.

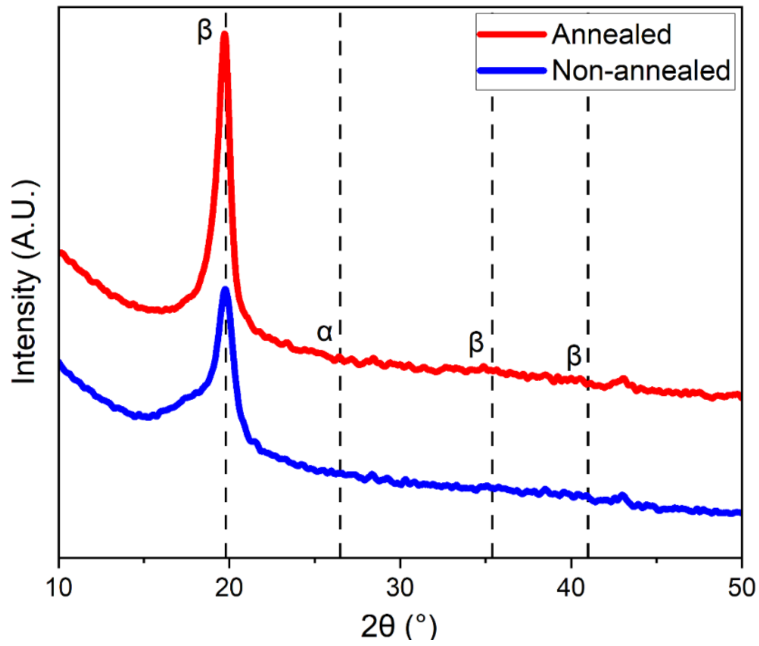

Fig. S2 XRD comparison of the diffraction spectra of the annealed and the non-annealed P(VDF-TrFE) nanofibers to confirm the enhanced crystallinity of the piezoelectric polymeric material resulting from the thermal annealing process. The graph compares an annealed sample (red line) with a non-annealed sample (blue line). The annealing process increased the crystallinity of the material from approximately 74.1% to 84.7%, confirming a successful contribution of the thermal annealing process to the piezoelectric polymeric material.

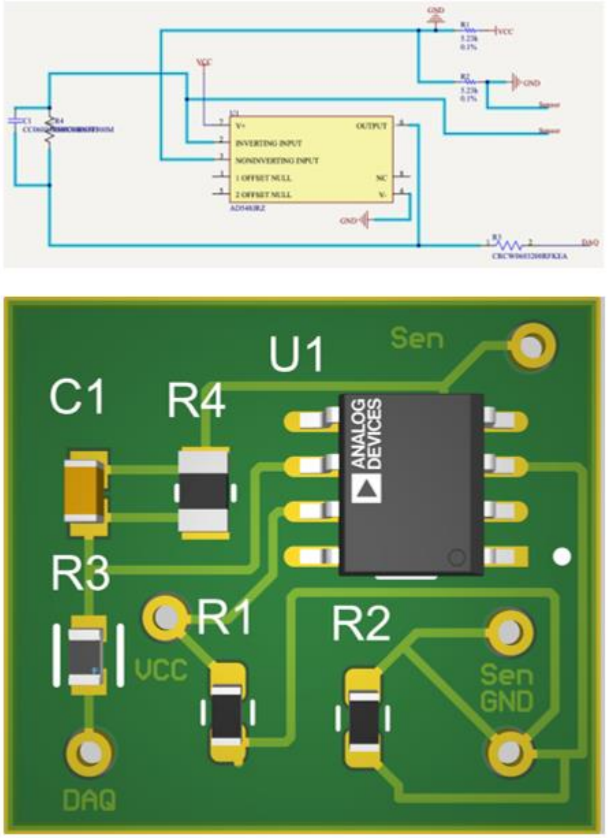

Fig. S3 Diagram of the charge amplifier circuit (top) depicting connections between the charge amplifying chip, capacitors, and resistors, along with the PCB diagram (bottom) used for the on-body signal processing of the flexible PCB. Altium was used to generate the two diagrams.

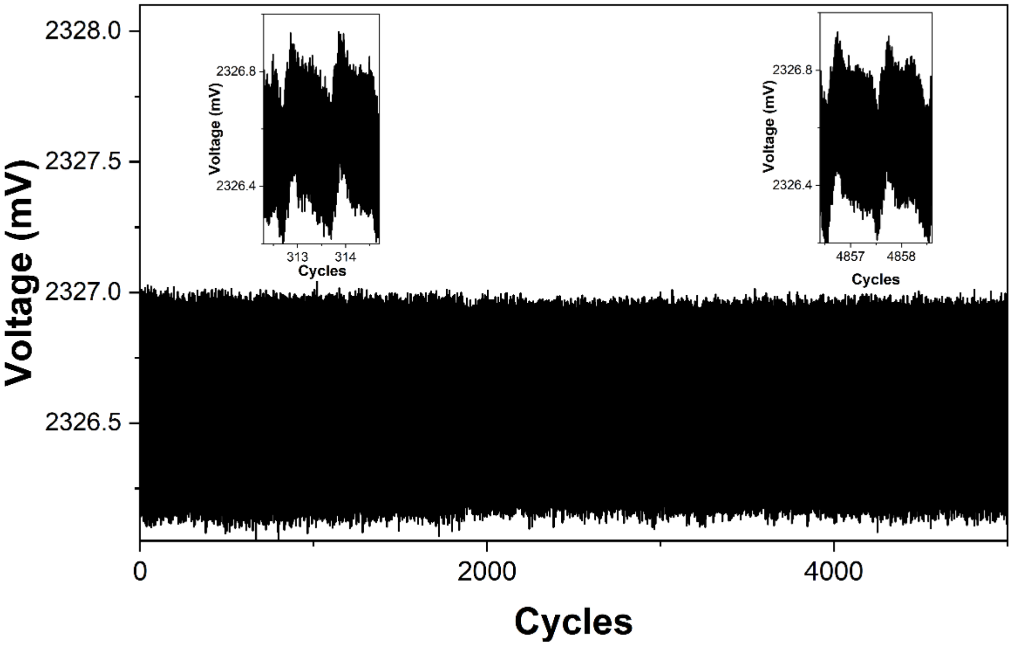

Fig. S4 Anti-fatigue testing at cyclic bending for 5,000 cycles to assess the performance stability of the performance stability of the annealed piezoelectric sensor. The beginning and ending cycles (left and right insets) show negligible loss in piezoelectric response to cyclic bending even after 5,000 cycles, demonstrating the stability of the P(VDF-TrFE) sensing layer and the CNT-based flexible electrode layers under repeated stress.

Table S1. The tabulated P1 and P2 values from twelve radial pulse waveforms. The augmentation index, which is a well-established measure of systemic arterial stiffness, is also calculated by taking the ratio of P2 and P1, demonstrating an average of approximately 0.55 and a standard deviation of 0.04.

Point	T(sec)	P1	P2	P2/P1
1	0.428	1.42E-03	4.09E-04	0.580947
2	1.18933	1.05E-03	5.65E-05	0.579019
3	1.97433	1.38E-03	4.27E-04	0.619586
4	2.78433	1.35E-03	3.55E-04	0.526991
5	3.577	1.45E-03	2.36E-04	0.508379
6	4.37933	1.08E-03	2.31E-04	0.537086
7	5.188	1.15E-03	3.22E-04	0.560846
8	5.98633	1.18E-03	3.88E-04	0.562831
9	6.78533	9.96E-04	3.24E-04	0.560636
10	7.58733	1.09E-03	3.03E-04	0.499529
11	8.39267	1.16E-03	1.22E-04	0.481502
12	9.20067	9.61E-04	1.17E-04	0.582071
			**Average**	**0.55**
			**Standard Deviation**	**0.04**

Table S2. The tabulated values of the isovolumetric contraction time (IVCT) and left ventricular ejection time (LVET) from eight consecutive cardiac SCG cycles. The IVCT yielded an average of 64 ± 3 ms, and the LVET averaged 335 ± 16 ms.

	IVCT (ms)	LVET (ms)
1	60	330
2	65	333
3	62	317
4	64	334
5	66	366
6	67	319
7	63	349
8	67	333
**Average**	**64**	**335**
**Standard deviation**	**3**	**16**

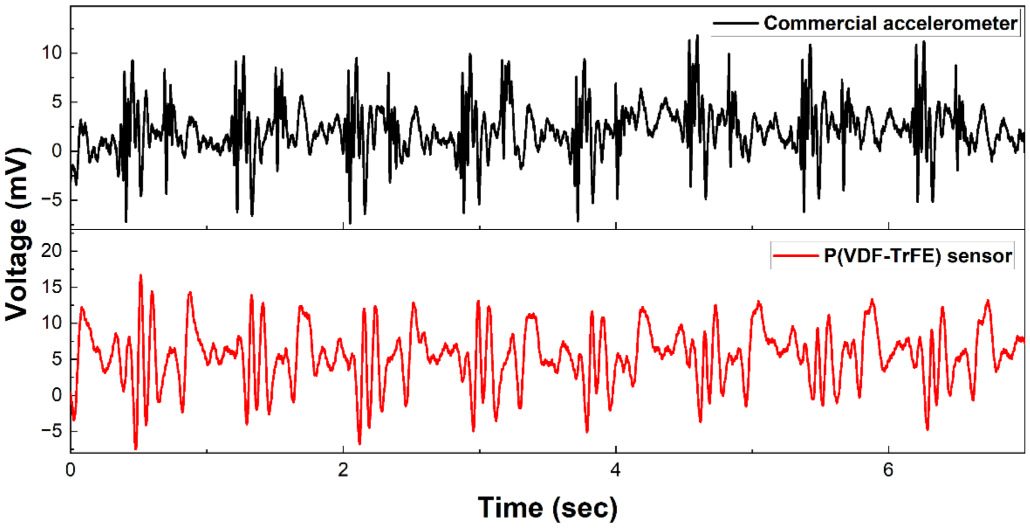

Fig. S5 Comparison graph between the commercial accelerometer (black) and P(VDF-TrFE) sensor (red) when collecting SCG signals from a volunteer's chest. Voltage outputs between both devices show comparable SCG waveforms, demonstrating high accuracy and reliability.

Video S1. Real-time Wrist Pulse Signal Acquisition.

The video demonstrates the monitoring of peripheral arterial dynamics from a healthy volunteer at rest, in which the integrated wearable system consisting of the flexible piezoelectric sensor and the on-body flexible PCB is positioned directly over the radial artery. The real-time electrical signals, conditioned by the flexible PCB, are displayed on the monitor. The clear, periodic waveform represents the radial artery pulse, demonstrating the system's effectiveness in capturing processed and detailed peripheral pulse signals.

Video S2. Real-time Carotid Pulse Signal Acquisition.

The video demonstrates the acquisition of the carotid artery pulse, a high-fidelity proxy for central aortic pressure, in which the integrated system is placed on the side of the volunteer's neck. The monitor shows the real-time detailed carotid pulse waveform, characterized by its distinct systolic and diastolic features, confirming the system's ability to process and monitor central hemodynamics.

Video S3. Real-time SCG Signal Acquisition.

The video shows the acquisition of SCG signals, in which the flexible integrated system is attached to the center of the volunteer's chest. The monitor displays the real-time, high-fidelity SCG waveform, which corresponds to the subtle vibrations of the chest wall produced by the mechanical events of the cardiac cycle. This demonstrates the system's capability to non-invasively capture detailed information on direct cardiac mechanical function in real-time.

Experimental Methods:

Preparation of the P(VDF-TrFE) solution. A polymer solution was prepared by dissolving 18 wt% P(VDF-TrFE) powder (70/30 mol ratio; Arkema) in a 1:1 volume ratio of N,N-Dimethylformamide (DMF) and Acetone (Sigma-Aldrich). The mixture was stirred under magnetic agitation for 6 hours at 50°C to ensure complete dissolution.

Preparation of the annealed P(VDF-TrFE) nanofibers. The P(VDF-TrFE) solution was electrospun using a commercial electrospinning unit (MTI Corporation). The process parameters were set to an applied voltage of 12 kV, a tip-to-collector distance of 16 cm, a mandrel rotating speed of 2000 rpm, and a solution feed rate of 1 mL/hr. The collected nanofiber mat was subjected to thermal annealing in a convection oven at 135 °C for 2 hours. This temperature was selected to be above the material's Curie temperature (~115 °C) and below its melting point (~150 °C) to promote the transition to the β-phase and enhance crystalline perfection.

Preparation of encapsulation layers and electrodes. The silicone elastomer base (Dow Chemical Company, Sylgard 184) was mixed with its curing agent at a 10:1 mass ratio to prepare the PDMS solution, which was subsequently degassed in a vacuum desiccator. Using a vacuum spin coater (MTI Corporation), the solution was spin-coated onto glass slides at 500 rpm for 30 seconds and partially cured in an oven at 60°C for 30 minutes to create semi-cured films. Separately, a CNT solution was prepared by ultrasonicating the suspension, which was then vacuum-filtrated onto a polytetrafluoroethylene (PTFE) membrane to create a CNT percolating mat. This mat was then transferred onto the partially cured PDMS substrates via contact pressing to form the electrode-coated encapsulation layers.

Device assembly. The final multi-layered device was assembled by carefully placing the annealed P(VDF-TrFE) fibrous membrane onto one of the CNT-coated PDMS layers. Before that, electrical wires are attached to the CNT electrodes using a conductive carbon adhesive. The second CNT-coated PDMS layer was then laminated on top to complete the sandwich structure.

Flexible PCB fabrication. For signal processing, the sensor was mounted onto a custom two-layer flexible PCB fabricated on a 25 μm polyimide dielectric with 1/3 oz electro-deposited copper traces, with the board measuring 12.57 mm × 14.99 mm with a total thickness of 0.11 mm. Surface finishes consisted of electroless nickel immersion gold with 1 μin gold thickness over a white silkscreen overlay, and an AD548 precision instrumentation amplifier chip served as the central footprint. The flexible PCB module was powered by a DC power supply (Keysight) at 15 V, and the collected electrical signals were collected via a data acquisition (DAQ) card system (National Instruments) through the LabView software on a computer.

Material and electromechanical characterization. The microscopic morphology of the electrospun nanofibers was observed using SEM (JEOL JSM-9700 F). The experiments for

crystalline phase identification of the P(VDF-TrFE) membrane before and after annealing were performed using XRD (PANalytical Empyrean) and FTIR (Agilent Gary 670). For the force and frequency response tests, the sensor was mounted on a shaker-based platform and subjected to cyclic compressive forces from a mechanical shaker (The Modal Shop) equipped with a load cell (PCB Piezotronics). The shaker was driven by a function generator (Keysight) connected to a power amplifier (The Modal Shop) to control the frequency and amplitude of the applied forces. The real-time electrical output generated by the device was collected and measured by an oscilloscope (Keysight) and LabView. The applied forces were separately measured by a load cell, and the data was collected via DAQ through LabView.

A note on the anti-fatigue test (**Figure 3E**): It is important to clarify a minor difference in the experimental setup for this long-duration test compared to the force-linearity tests (**Figure 3C**). To ensure mechanical stability over 10,000 cycles, the mounting fixture required a slightly larger initial distance between the actuator head and the device surface. Even though the applied force was calibrated to the same 5 N at 1 Hz, this subtle difference in the setup resulted in slightly lower effective stresses being transferred to the active piezoelectric layer. This accounts for the lower absolute voltage observed in the anti-fatigue test compared to the single 5 N measurement in **Figure 3C**. The primary purpose and key finding of this test is the demonstration of the sensor's exceptional signal stability and durability over a prolonged period, rather than a direct comparison of absolute voltage magnitude.

References

[1] Ruan, L.; Yao, X.; Chang, Y.; Zhou, L.; Qin, G.; Zhang, X.. Properties and Applications of the β Phase Poly(vinylidene fluoride),. Polymers (Basel) 2018, 10 (3). DOI: 10.3390/polym10030228.
